# Segmental Defect-Bridging Intramedullary Knee Arthrodesis for Osseous Hydatidosis of the Distal Femur: A Case Report

**DOI:** 10.7759/cureus.13273

**Published:** 2021-02-11

**Authors:** Enejd Veizi, Ahmet Fırat, Şahin Çepni, Hacı M İnan, Kasım Kılıçarslan

**Affiliations:** 1 Orthopedics and Traumatology, Ankara City Hospital, Ankara, TUR; 2 Pathology, Ankara City Hospital, Ankara, TUR

**Keywords:** osseous hydatidosis, hydatid cyst, femur fracture, knee arthrodesis

## Abstract

Hydatid cyst is a condition endemic to many parts of the world and is mainly caused by *Echinococcus granulosus (E. granulosus). *It rarely affects the bone tissue, with the most commonly impacted sites being the vertebrae and the pelvis. Preoperative diagnosis is challenging and very rarely possible because of its similarities with other pathologies. In this report, we present the case of a 64-year-old patient with osseous hydatidosis of a pathological distal femur fracture. The fracture pattern was not recognized on the initial operation and multiple serial debridements were required to control the disease, leading to a large bone defect and a weakened extensor mechanism. A knee arthrodesis with a segmental defect-bridging intramedullary system was eventually performed, which led to satisfying outcomes.

Osseous hydatidosis very often presents itself as a pathological fracture and is difficult to diagnose preoperatively with plain radiographs. Orthopedic surgeons are advised to maintain a high index of suspicion and to test for this disease when cystic bone lesions are detected at fracture sites, especially in patients from endemic regions.

## Introduction

Echinococcosis is a zoonotic disease endemic to the Mediterranean basin, Latin America, Africa, some districts of Australia, with notable morbidity reported from some parts of Asia as well [1]. It is caused by a cestode, more specifically by *Echinococcus granulosus (E. granulosus)* in most cases. Once it gains entry into the human body, it spreads hematogenously and generally afflicts the lungs and the liver [2].

Although osseous involvement in this condition amount to less than 1% of all cases and is frequently secondary to hepato-pulmonary hydatidosis, primary cases have also been reported [3]. At early stages, the lesions tend to be asymptomatic and present only in locally advanced stages with pain, deformity, and pathological fractures. Preoperative diagnosis is challenging and very rarely possible because of its similarities with other pathologies. Despite the fact that there have been numerous case reports from various regions on this topic, a definitive consensus has not been reached on a treatment protocol for such cases [4].

In this report, we discuss the case of a 64-year-old female patient with a pathological distal femur fracture who underwent numerous surgeries and was ultimately treated with a segmental defect-bridging intramedullary knee arthrodesis system.

## Case presentation

A 64-year-old female patient presented to the emergency room with pain in her right distal thigh. She had a history of a minor fall. Physical examination revealed a swollen, painful right thigh. Except for a BMI of 31, the patient had no other known comorbidities and was under no medications. She lived in a rural area far from the capital.

**Figure 1:**
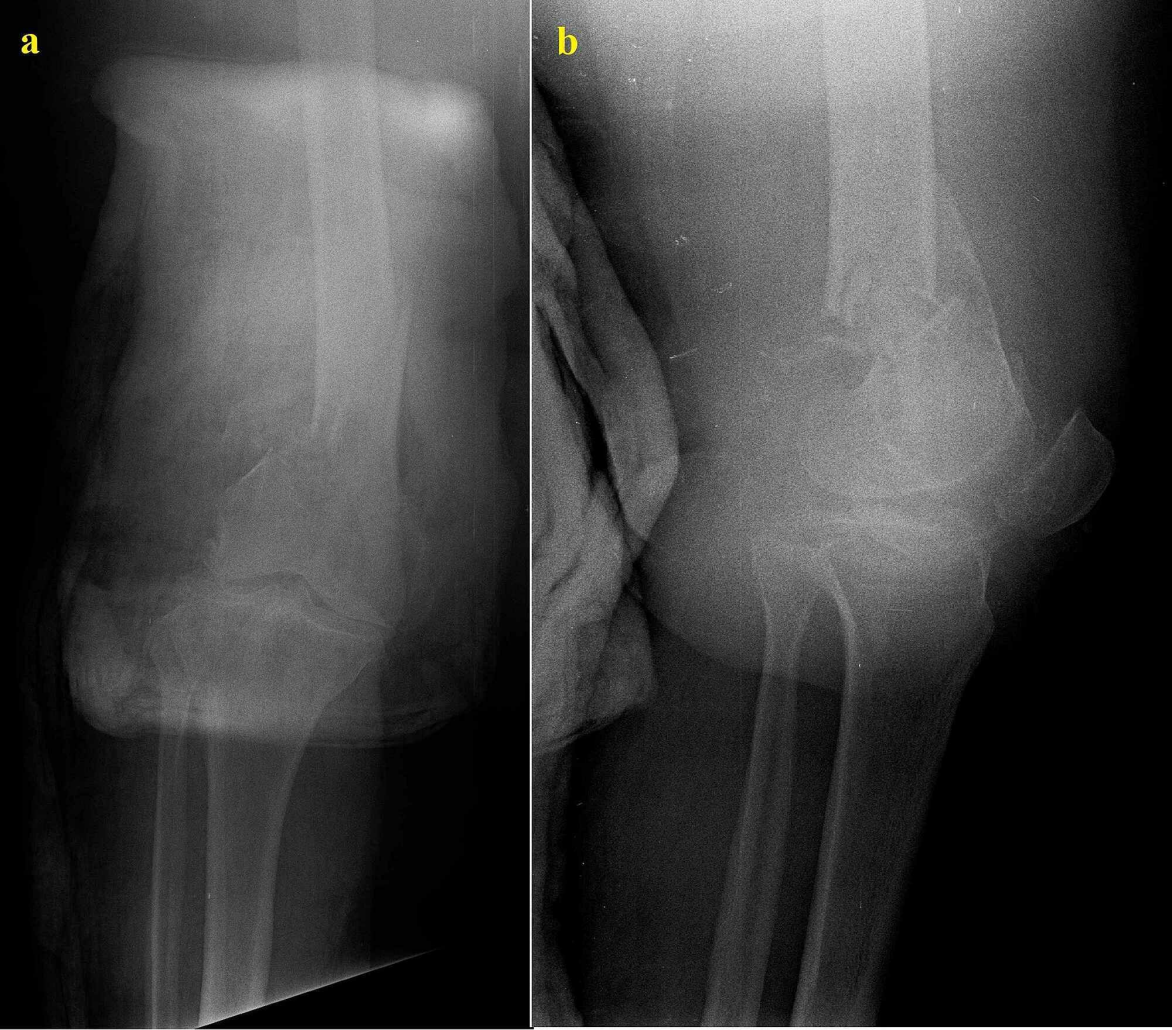
Initial anteroposterior (a) and lateral (b) radiographs of the patient showing the distal femoral fracture At closer observation, the cystic/lytic lesion can already be seen

**Figure 2:**
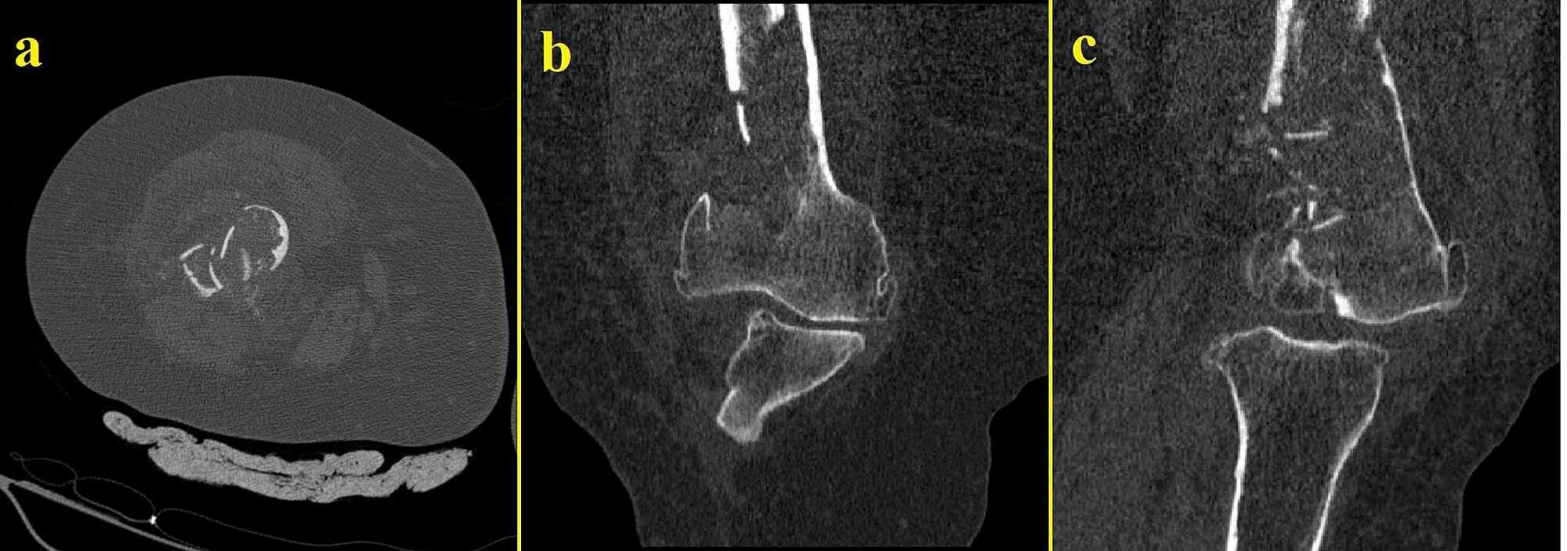
Initial CT scans of the same extremity taken on the day of trauma Thinning of the cortexes and lysis can be observed CT: computed tomography

She was eventually diagnosed with a distal femoral fracture (Figure 1). Preoperative blood tests were normal with no elevation of C-reactive protein (CRP) or white blood cells detected. The fracture was reduced and internally fixed with a minimally invasive technique. The patient was discharged two days later. The surgery was performed by a junior surgeon who failed to recognize the initial nature of the fracture despite the CT scan (Figure 2).

**Figure 3:**
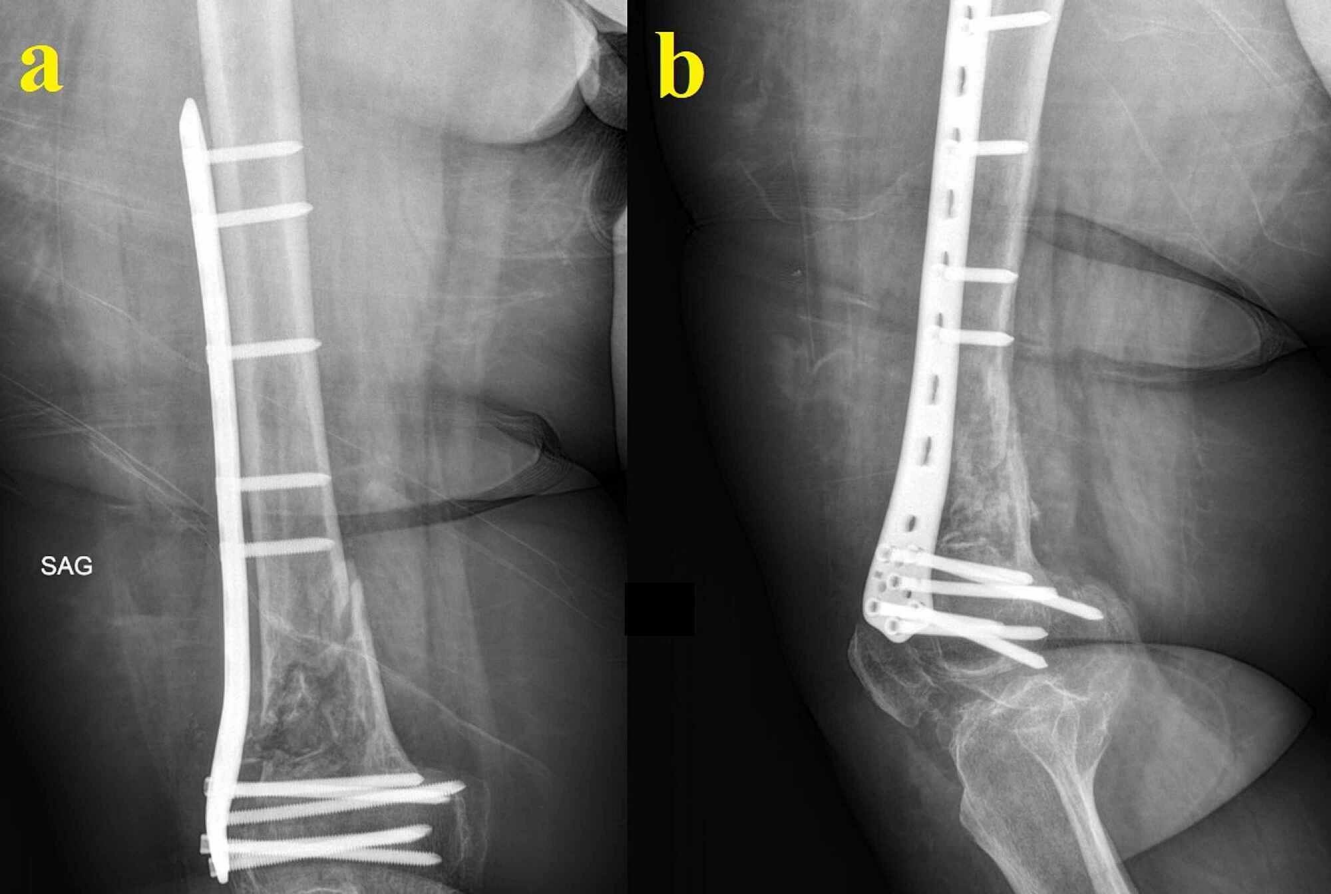
Radiographs in the second month postoperatively The patient has pain on ambulation. The radiographs show no signs of healing and reveal lysis and resorption around the fracture site

Initial follow-ups were carried out by her GP. The patient described persistent pain at the operation site. She was able to be mobile indoors only. Her radiographs in the second month postoperatively (Figure 3) showed no signs of healing and revealed lysis and resorption around the fracture site.

**Figure 4:**
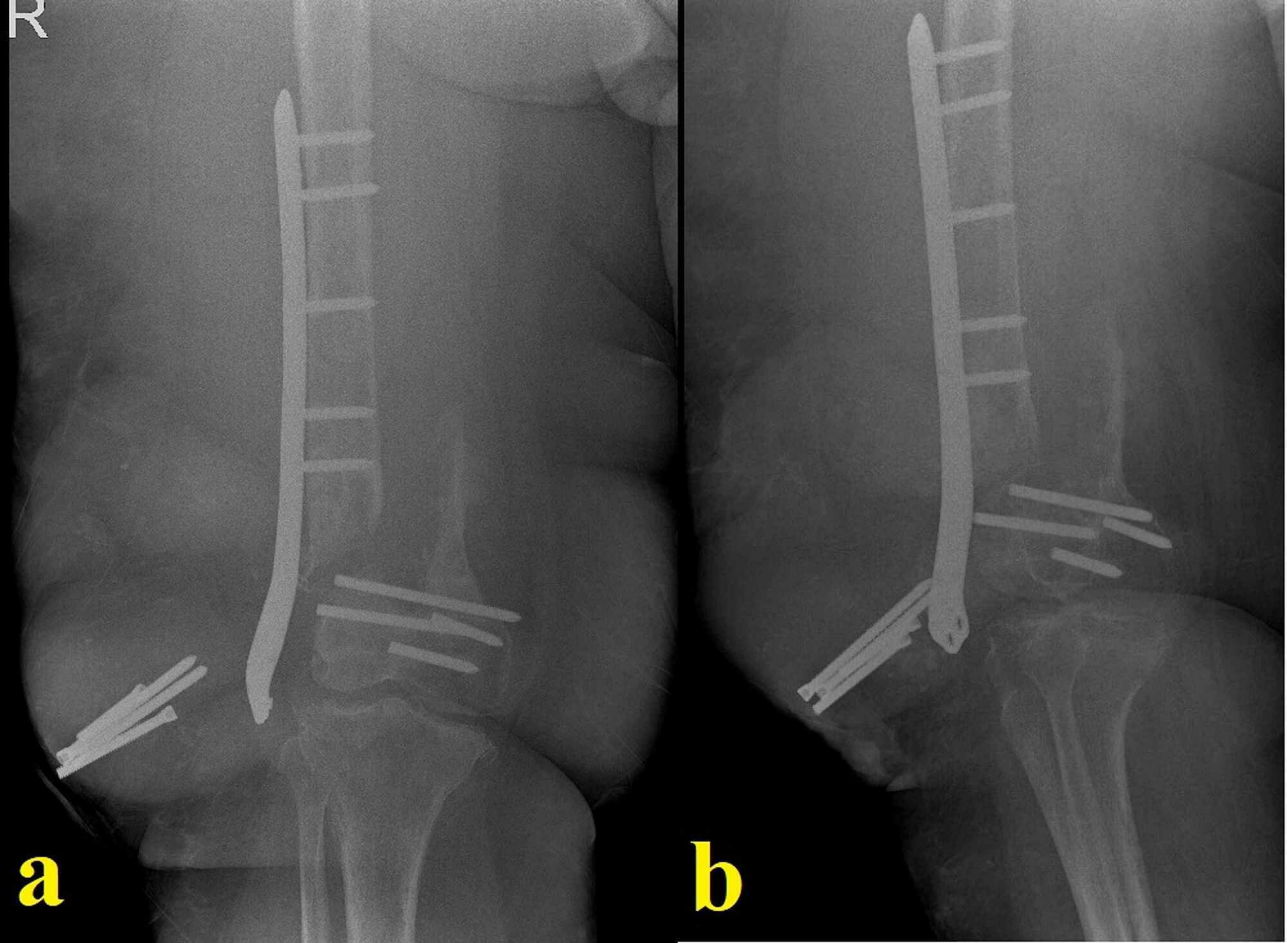
Radiograph at six months postoperatively The image shows failure at the fracture site with no signs of callus, resorption, thinning of the cortexes, and multiple failed distal screws

A biopsy was scheduled to be performed for a differential diagnosis including osteomyelitis, benign cystic lesion, giant cell tumor, fibrous dysplasia, and neoplastic lesions/metastases. However, the patient did not consent to a biopsy and only a CT scan of the bone was performed, which revealed thinning of the cortexes, no callus on the fracture site, and lysis. The patient was informed of the possible outcomes. At six months postoperatively, she showed up at the outpatient clinic with the same swollen thigh, pain on palpation, and inability to bear weight. Her radiograph at that time showed extensive lysis and displacement of the fracture site with multiple failed distal screws (Figure 4).

**Figure 5:**
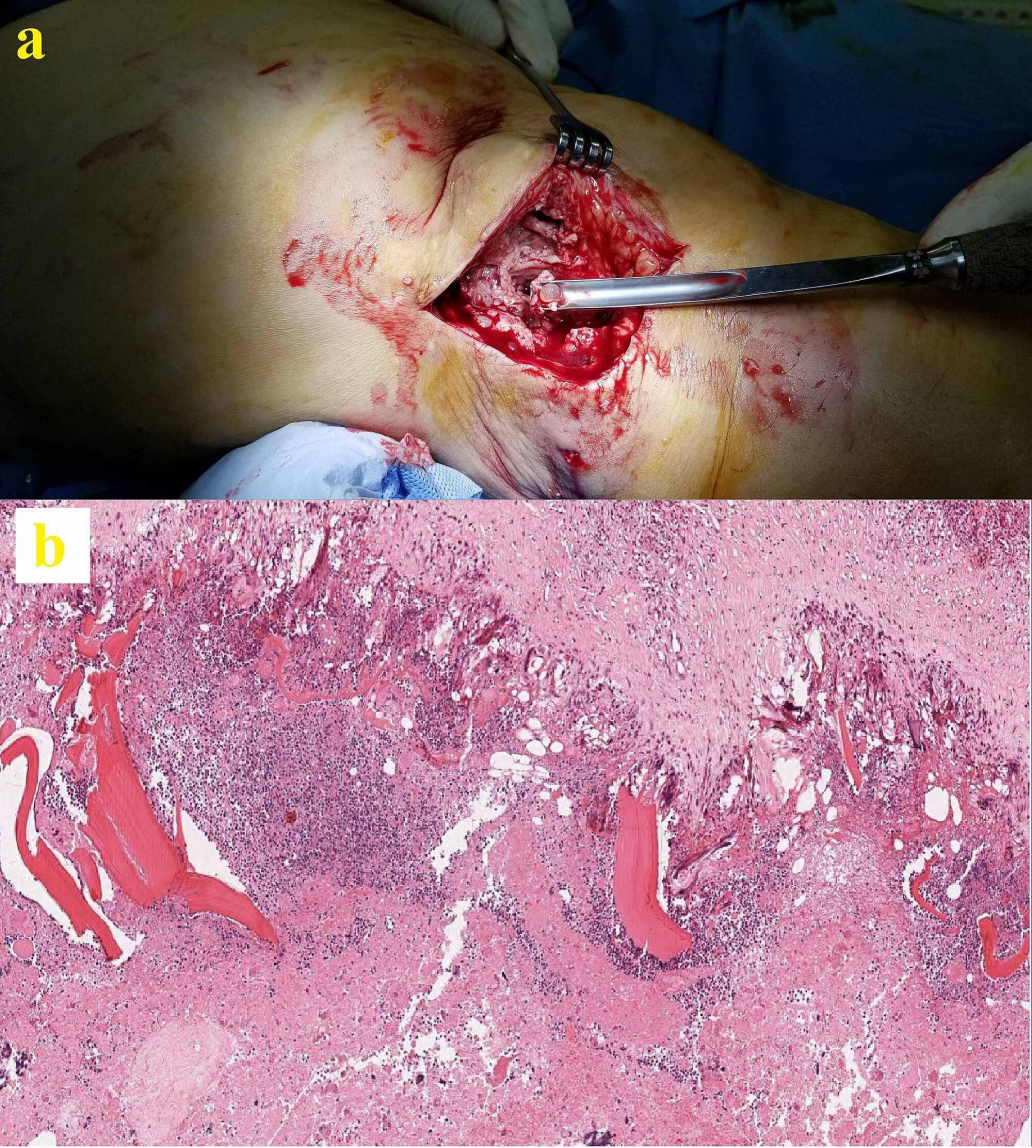
One of many pearly-white cysts excised and debrided during surgery (a) and its histopathological examination with hematoxylin and eosin stain (b)

Multiple cystic lesions were present at and around the fracture site. The patient was taken to the operating room; a thorough debridement, sequestrectomy, and saucerization were performed. We also observed and removed multiple pearly-white cystic entities throughout the affected bone and surrounding soft tissue (Figure 5a). The metaphyseal-diaphyseal part of the femur was the most affected part, but the articular surfaces were found to be intact. Samples were taken for a histopathological examination. We removed all implants and irrigated the site with saline and iodine solutions. A block of custom-shaped polymethyl methacrylate (PMMA) was placed on the remaining osseous structures and the wound was closed.

The histopathological exam revealed cuticular membrane fragments-containing cyst wall with inflammatory reaction in the presence of giant cells (Figure 5b). Scolices of *E. granulosus* with a chain of hooklets in the germinal layer were also present. The patient was started on albendazole 15 mg/kg/day, which was to be continued for six months. The patient was treated in a multidisciplinary manner, and we consulted with the departments of Infectious Diseases and General Surgery. She underwent abdominal ultrasonography and cranial, vertebral, thoracic, and abdominal CT scans, as well as multiple radiographs to rule out possible liver or lung cysts. A consensus was reached on the fact that this was an isolated lesion of the distal femur and no cysts were detected on other sites.

Four months later, the patient presented with discharge from the wound site. She underwent two debridement sessions, and the wound was thoroughly debrided. Suspecting a superinfection, ciprofloxacin and rifampin were added to the initial albendazole. Despite the cultures coming back negative, the triple therapy was continued for a period of three months.

**Figure 6:**
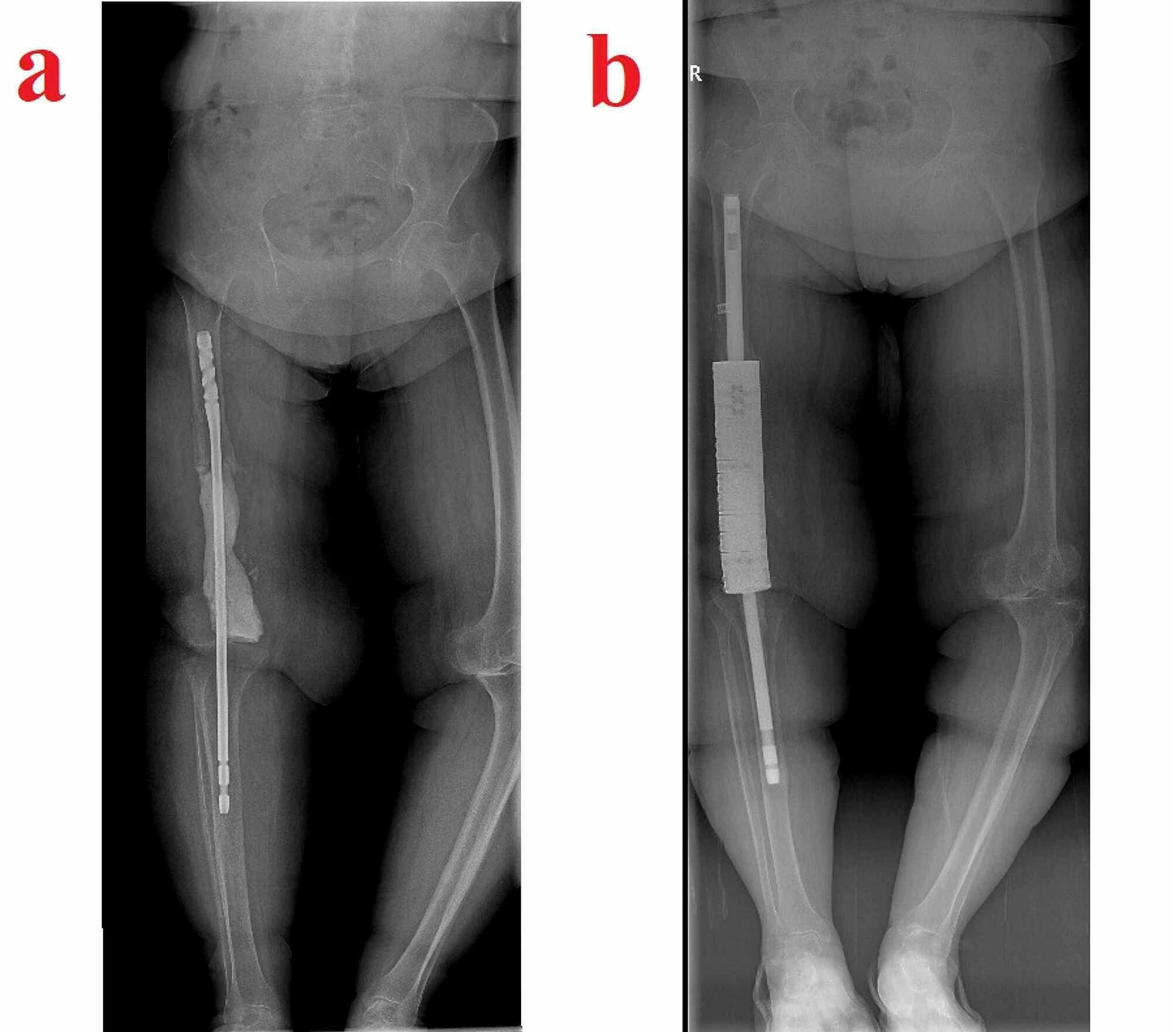
Images related to temporal arthrodesis and final standing radiograph The temporal arthrodesis performed after extensive segmental resection using a long knee-spanning femoral nail centrally covered with a PMMA block (a) and the final standing radiograph showing the segmental defect-bridging intramedullary knee arthrodesis system chosen for the case (b) PMMA: polymethyl methacrylate

Six months after the last operation, the patient presented again with serous discharge. Her general and mental status had suffered greatly due to the continuous interventions. She was anemic and in a state of depression. This time, we opted for a more aggressive debridement. All of the remaining distal femur together with a portion of the diaphysis and the indurated surrounding soft tissue was removed. The extensor mechanism had suffered from the repeated surgeries but was intact and was thus preserved. Both the femoral and the tibial intramedullary canals were reamed open, and an arthrodesis was performed using a long knee-spanning femoral nail centrally covered with a PMMA block (Figure 6a). Hypertonic saline and iodine solutions were used for wound washing. The wound was closed uneventfully, and the patient was started again on albendazole. She was allowed to be mobile with a cane and weight-bearing was restricted for the first two months. She was advised at this point that because of the extensive debridement procedures and infected bone removal, an amputation might be needed as a salvage procedure in the event of a lack of healing. A definitive arthrodesis or, less likely, a condylar prosthetic was also discussed in case of healing.

One year after the last operation, the patient finally had a clean wound. She was able to be mobile indoors with the help of a cane and was determined to undergo a final decisive procedure. Her physical examination showed a loss of strength in her right lower extremity and an atrophic quadriceps muscle. CRP and erythrocyte sedimentation rate were normal. There was no discharge sinus. Her case was discussed with other senior surgeons and a definitive arthrodesis procedure was planned. Long radiographs showed a bone defect of 22 centimeters compared to the healthy left side, and a segmental defect-bridging intramedullary knee arthrodesis system was chosen for the procedure (Figure 6b). A frozen section came back clean and the arthrodesis was performed without complications. She is now in her 16th postoperative month and can be mobile with full weight-bearing. Yearly checkup visits were recommended.

## Discussion

Hydatid disease is commonly caused by *E. granulosus*, a parasitic tapeworm that uses canines as definitive hosts [2]. While *Echinococcus multilocularis (E. multilocularis)* is known to cause alveolar hydatid and is very rare in humans, *E. granulosus* uses cattle as intermediate hosts, and humans are infected by ingestion of food that is contaminated by fecal matter of the definitive hosts [2]. Humans are accidental and dead-end hosts and are commonly infected due to the consumption of unwashed vegetables [5]. The adult forms of the larva reside in the small intestine of the canine and release eggs in the feces. These eggs are ingested by the intermediate hosts and the eggs hatch in the intestine. Oncospheres that have the capacity to penetrate the intestinal wall are released and the parasite gains entry to the circulatory system. Because the liver and lung both share high vascularity and filtrating traits, the oncospheres are mostly entrapped here. They develop into cysts that produce protoscolices and daughter cysts. In rare cases, the oncospheres are able to pass through these organs and that is how extrapulmonary and extrahepatic infestation occurs. Other target organs include the kidney, spleen, eye, and central nervous system [6].

It is a condition characterized by a high treatment cost and non-standardized procedures, all aimed at eradicating an otherwise resistant condition. Even in endemic regions, the bone involvement is below 1% of all cases [5]. The thoracic vertebrae seem to be the most commonly infected region, followed by the lumbar vertebrae, the pelvis, femur, and humerus [6]. Most of these lesions are solitary (83%) [4]. Because bone tissue, and especially the cortex, is a hard structure, the cyst faces a lot of resistance. It is mostly located in the spongious bone where it slowly but surely enlarges itself, causing chronic ischemia. Ischemia leads to vascular impairment and lytic lesions and erosion and thinning of the cortexes, which result instead in pathological fractures [1,5].

Diagnosis is mostly impossible preoperatively and most cases require a histological analysis [4]. No pathognomonic signs exist, and radiological signs can be confusing. Direct X-rays, CT, and MRI are the preferred modalities for diagnosis. While CT is the investigation of choice for the analysis of the extent of the bone involvement, MRI is a good method to detect recurrences and soft tissue involvement [7]. Plain X-rays, the most frequently used early diagnostic tool, show multiloculated cysts without sclerosis or calcification [7]. Differential diagnosis should include chronic osteomyelitis, benign cystic lesion, giant cell tumor, fibrous dysplasia, brown tumor (hyperparathyroidism), and various other neoplastic lesions [3], bone metastases, and intraosseous ganglion [8]. Histological examination of the surgical specimen remains a must.

There is little consensus on the treatment of these bony lesions [4]. Most authors seem to prefer a combined surgical and medical approach. The literature mentions palliative treatment with benzimidazoles of different dosages [9], amputations [1], debridement [4], drainage, laminectomy, arthrodesis [4], hemipelvectomy, and fixations with multiple surgical interventions, which are required in most cases [3,4]. Hypertonic saline and iodine-based solutions have been used to maximize the impact of washing and debridement, but their efficacy is still a matter of debate [9,10]. Bone graft augmentation and PMMA usage as a temporary and occasionally final procedure have also been suggested [11]. In our case, after a series of debridement surgeries that led to extensive muscle hardening and weakness, we opted for knee arthrodesis. Due to the extensive bone loss, a segmental partially cemented defect-bridging system with roughened titanium on both intramedullary ends, as often used in oncological cases, was employed as the treatment of choice. This ensured lower extremity length and extension mechanism preservation, giving the patient a chance for healthier mobility [12].

Due to the aggressive nature of the surgeries required and the nature of the cysts themselves, many potential complications have been mentioned in the literature. Anaphylactic reactions, although rare, are a potential risk in cases of cyst breakage [13]. Secondary bacterial surgical site infections or bedsore wound infections are a potentially deadly complication. The most frequently isolated organisms are *staphylococci*, *Escherichia coli,* and *Pseudomonas aeruginosa* [14]. Antibiotic usage for a period of six months to two years has been reported with some cases, leading to a higher treatment cost and longer hospital stays [4]. Even complete disease eradication seems very difficult and the rate of long-term local recurrence risk of bone echinococcosis is 17% [15]. Despite thorough debridement, daughter cysts left in situ after breakage of the original one can contribute to recurrence over time and may be difficult to treat without surgery. Complete excision of the lesion has been reported in only 16% of the cases, with amputations being the treatment of choice occasionally [16]. Steinmetz et al. [4], in their literature review of 200 cases, found distant organ involvement to be the only variable, and thus a risk factor, associated with local recurrence. In the presence of concomitant bone and visceral organ infestation, the incidence was five times greater for local bone recurrence or progression of disease in the bone. However, they were unable to establish whether these recurrences were truly local or reinfections from other organs. Other frequent complications include persistent pain, fractures, paraplegia, handicap, and pseudoarthrosis [4].

Segmental defect-bridging intramedullary systems, while still presenting a challenge for surgeons, have been used in oncological orthopedic surgery for a long time, and they offer lasting and relatively safe solutions for major bone defect cases where reconstruction with bone grafts or distraction osteogenesis techniques are not viable options [12]. They are generally used for oncological diaphyseal defects and very rarely for articular defects [12]. Other advantages include preservation and early motion of adjacent joints and survival for the lifespan of the patient [17,18]. Our patient was able to be mobile very soon after surgery with range of motion exercises in the adjacent joints and was left non-weight-bearing for three months. No complications have occurred up until the time of this report, at 16th month postoperatively.

Lessons learned 

We are aware of the shortcomings of our case, have learned from our mistakes, have taken measures to avoid repetition of the same in the future, and would like to share our learnings here. We learned that every 'atypical' or relatively low-energy fracture mandates an intraoperative pathological examination. In suspected cases, having a frozen section analyzed and receiving a negative result is always better than misdiagnosing a pathological fracture and having to deal with complications. Since ours is a knowledge-based, but most importantly, experience-based profession, we learned that atypical fractures can occasionally be misjudged by junior and less experienced surgeons. To make up for this temporary lack of experience and to avoid repetitions of such situations in the future, we now use online in-house social media groups to share trauma cases that apply to our center. Deprived of individual patient information, X-rays, and sometimes CT scans, are shared by all physicians on duty (junior or senior), and proactive discussions on relative treatment options that this practice generated have led to a very robust and prolific scientific environment.

## Conclusions

Osseous hydatidosis is a relatively rare and difficult-to-treat condition affecting individuals of all ages. It presents itself as a pathological fracture very often and is difficult to diagnose preoperatively with plain radiographs. Orthopedic surgeons are advised to maintain a high index of suspicion and to test for this disease when cystic bone lesions are the cause of such fractures, especially in patients from endemic regions. In patients with large bone defects, among medical and other surgical treatment options, segmental defect-bridging intramedullary systems should be considered during preoperative planning.
